# A Novel Clinical Nomogram for Predicting Cancer-Specific Survival in Adult Patients After Primary Surgery for Epithelial Ovarian Cancer: A Real-World Analysis Based on the Surveillance, Epidemiology, and End Results Database and External Validation in a Tertiary Center

**DOI:** 10.3389/fonc.2021.670644

**Published:** 2021-04-20

**Authors:** Xianli Li, Haoya Xu, Limei Yan, Jian Gao, Liancheng Zhu

**Affiliations:** Department of Obstetrics and Gynecology, Shengjing Hospital of China Medical University, Shenyang, China

**Keywords:** epithelial ovarian cancer, prognosis, nomogram, SEER database, cancer-specific survival

## Abstract

**Background:**

The present study aimed to construct and validate a nomogram that can be used to predict cancer-specific survival (CSS) in patients with epithelial ovarian cancer (EOC).

**Methods:**

A total of 7,129 adult patients with EOC were extracted from the Surveillance, Epidemiology, and End Results database between 2010 and 2015. Patients were randomly divided into the training and validation cohorts (7:3). Cox regression was conducted to evaluate prognostic factors of CSS. The internal validation of the nomogram was performed using concordance index (C-index), AUC, calibration curves, and decision curve analyses (DCAs). Data from 53 adult EOC patients at Shengjing Hospital of China Medical University from 2008 to 2012 were collected for external verification. Kaplan–Meier curves were plotted to compare survival outcomes among risk subgroups.

**Results:**

Age, grade, histological types, stage, residual lesion size, number of regional lymph nodes resected, number of positive lymph nodes, and chemotherapy were independent risk factors for CSS. Based on the above factors, we constructed a nomogram. The C-indices of the training cohort, internal validation cohort, and external verification group were 0.763, 0.750, and 0.920, respectively. The calibration curve indicated good agreement between the nomogram prediction and actual survival. AUC and DCA results indicated great clinical usefulness of the nomogram. The differences in the Kaplan–Meier curves among different risk subgroups were statistically significant.

**Conclusions:**

We constructed a nomogram to predict CSS in adult patients with EOC after primary surgery, which can assist in counseling and guiding treatment decision making.

## Introduction

Among malignant gynecological tumors, ovarian cancer (OC) ranks third in incidence and first in mortality rate. A recent study estimated that there were over 20,000 new cases of OC and over 13,000 deaths due to OC in the United States in 2020 (1).

Epithelial ovarian cancer (EOC) is the most common type of OC, accounting for 90% of OC cases, and the majority of EOC occurs in adults (2). The standard treatment for EOC is a combination of surgery and chemotherapy. Even so, most patients with EOC present at stage III (37%) or IV (28%) at the time of diagnosis, resulting in poor prognosis. The 5-year cancer-specific survival (CSS) rates for stage III and IV EOC are only 41% and 20%, respectively (3). In addition, due to the various factors that may affect cancer progression, evaluation of cancer prognosis based on American Joint Committee on Cancer (AJCC) stage alone is unpredictable (4). Therefore, it is of primary importance to establish an assessment system to guide prognostic evaluation for EOC and adjustments in specific treatment strategies.

Studies have demonstrated that absence of pelvic pain at diagnosis, International Federation of Gynecology and Obstetrics (FIGO) stage IIIC, suboptimal cytoreduction, presence of postoperative complications, inadequate adjuvant treatment, and pathological type of clear-cell cancer are prognostic factors for overall survival (OS) in patients with OC (5). However, to date, no comprehensive evaluation systems have been developed for determining postoperative prognosis in adult patients with EOC.

Among the most widely used prediction tools is the nomogram, which can be used to quantify risk and evaluate prognosis in patients with various types of cancer (6–10). Recent studies have indicated that the nomogram is superior to the AJCC staging system in predicting survival in patients with cancer (11–16). However, to our knowledge, there are currently only two nomograms that predict EOC prognosis in adults, and the sample sizes in the original studies were very small (17, 18). Therefore, it is of great clinical significance to immediately establish a large sample-based nomogram for predicting prognosis in adult patients with EOC.

In the present study, we aimed to analyze independent prognostic factors and construct a nomogram for predicting prognosis in adult individuals with EOC using data from the Surveillance, Epidemiology, and End Results (SEER) database of the National Cancer Institute (NCI). This study was externally validated in a cohort of patients with EOC treated in the Department of Gynecology at Shengjing Hospital of China Medical University. Our findings may aid clinicians in assessing patient outcomes more accurately and provide a foundation for patients with EOC to select individualized treatment.

## Materials and Methods

### Ethics Approval and Informed Consent

Informed patient consent was not needed for the *SEER* database data, as cancer is a publicly reportable disease in every state in the United States.

Ethical approval for the use of patient data for external validation in this study was obtained from the Clinical Research Ethics Committee of Shengjing Hospital of China Medical University (Approval No. 2020PS533K), and all patients provided signed informed consent in accordance with the Declaration of Helsinki.

### Data Source and Extraction

Patient data for the current study is obtained from the *SEER* database, which is one of the most representative large tumor registration databases in North America, including data from 18 cancer registries and covering 34.6% of the population of the USA (19). The *SEER* database has a large sample size and relatively complete follow-up information. EOC cases were retrieved from *SEER* database using SEER*Stat software version 8.3.6 (https://seer.cancer.gov/seerstat/) (account ID: 19731-Nov2019).

Detailed selection of EOC patients in 2010-2015 from SEER database. The inclusion site code was C56.9-Ovary, and the histological code was Serous: 8441, 8442, 8460, 8461, 8462, 8463, 9014; Mucinous: 8144, 8384, 8470, 8471, 8472, 8480, 8481, 8482; Endometrioid: 8380, 8381, 8382,8383; Clear cell: 8310 and 8313, 8443, 8444, 9110; Transitional cell: 8120, 8122, 8130, 9000; Epithelial stromal: 8800,8801, 8804, 8805, 8810, 8814, 8840, 8850, 8851, 8854, 8890, 8891, 8896, 8900, 8901, 8902, 8920, 8921, 8930, 8931, 8933, 8935, 8936, 8950, according to the International Classification of Tumor Diseases, Third Edition (ICD-O-3).

Inclusion and exclusion criteria: (1) Patients with pathologically confirmed EOC were included, and patients with multi-source tumors and non-primary tumors were excluded. (2) Six types of EOC conforming to WHO (2014) were included (serous, mucinous, endometrioid, clear cell adenocarcinoma, transitional cell tumor [including malignant Brenner tumor and transitional cell carcinoma], and epithelial-stromal [including adenosarcoma and carcinosarcoma]) (20), and patients with unknown histological type (NOS patients) or could not be clearly classified into the above six histological subtypes were excluded. (3) including age, race, marriage, insurance factors, excluding age of patients under 19 years old. (4) Due to histological grade and stage are prognostic factors, histological grade and AJCC stage data were included, and patients with incomplete information above were excluded. (5) Patients who had undergone surgery for the primary lesion with clear surgical method, complete surgical records of lymph nodes, tumor size and residual lesions were included, and those who had not been operated were excluded. (6) Patients with complete follow-up information and cancer-specific death were included, and patients with incomplete follow-up time, other causes of death or unknown death status, and survival time for less than 1 day were excluded. A total of 7129 cases conforming to the screening criteria were included. In this study, the starting point of follow-up was the initial surgery for EOC, and the ending point was cancer-specific death or the end of follow-up was December 31, 2015.

There were 22 variables in this study, including year of diagnosis, age, race, insure, marriage, laterality, tumor size, preoperative serum CA125 level, surgery for primary lesions, regional lymph nodes dissected, histological grade, histologic types, AJCC stage, residual lesion size, lymph nodes positive, radiotherapy, neoadjuvant chemotherapy, chemotherapy, organ metastasis (bone, brain, liver, lung).

The clinical records of 53 patients who underwent surgery in the Department of Gynecology, Shengjing Hospital, China Medical University from 2008 to 2012 and were pathologically diagnosed as having EOC were retrospectively analyzed. Inclusion criteria: 1. Age ≥19 years old, surgery was primary surgery, the patients did not receive preoperative chemoradiotherapy or biological therapy; 2. The tumor was primary, and the postoperative pathological diagnosis was confirmed as EOC. The clinical data and postoperative follow-up data were complete. The cause of death was cancer-specific death. The end of follow-up was January 31, 2021.

### Statistical Analysis

**Figure 1:**
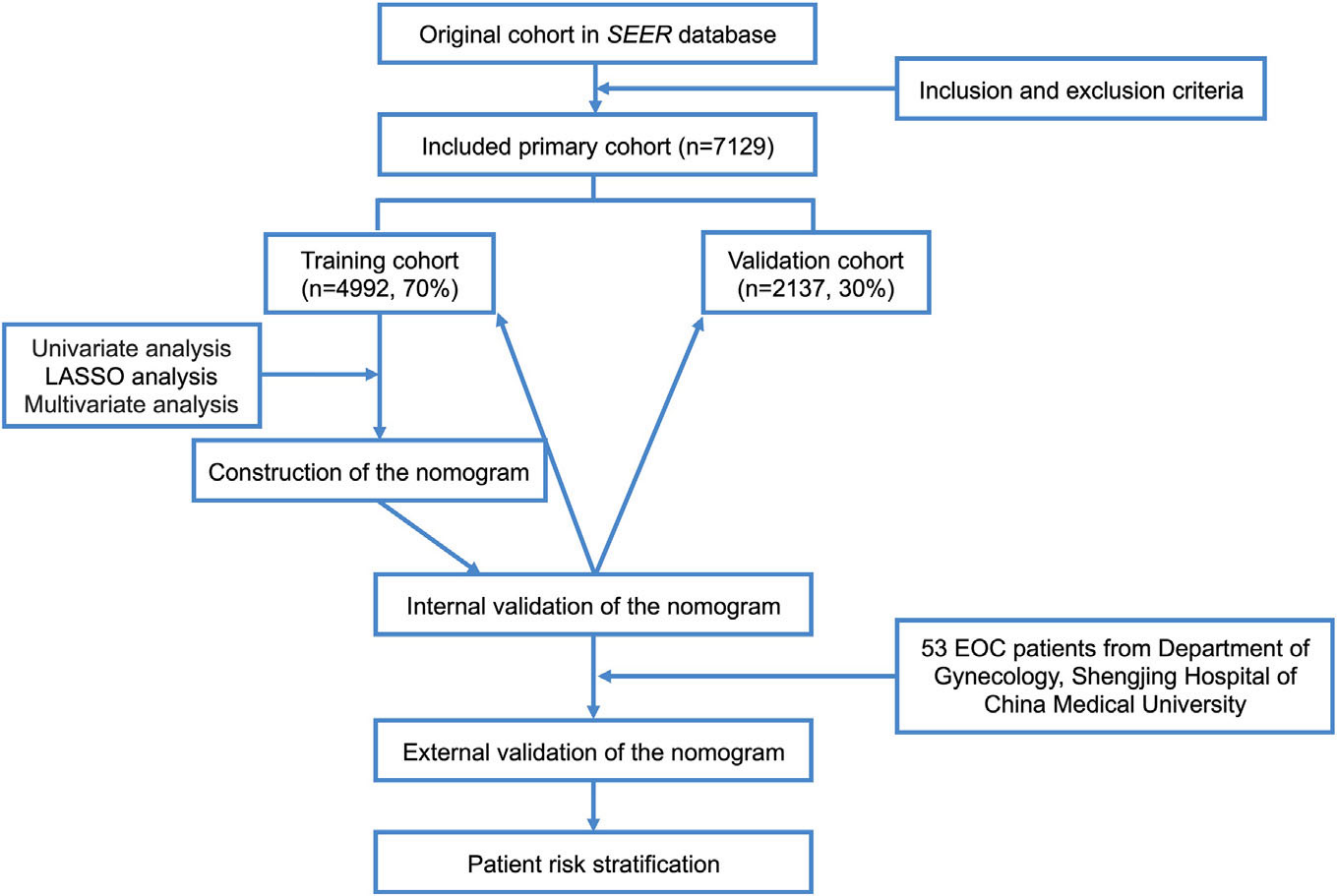
Flow chart of study procedures.

X-tile software v3.6.1 (Yale University, New Haven, Connecticut, USA) (21) was used to ascertain the optimal cut-off points for age, tumor size, and the number of positive lymph nodes. Patients enrolled in our study were randomized into the training cohort and validation cohort in a 7:3 ratio (22–24). Univariate analysis was performed using the log-rank χ2 test. Univariate variables with P values < 0.05 were included in the least absolute shrinkage and selection operator (LASSO) analysis, which was used to further select useful predictive features to avoid over-fitting to some extent. The results were then further incorporated into a Cox multivariate regression analysis. All independent prognostic factors in the Cox multivariate regression analysis (P<5.00e-05) were integrated, and a nomogram predicting CSS was constructed using R software version 3.6.0 (http://www.r-project.org/). We internally validated the model in the training cohort and the validation cohort separately. The Concordance Index (C-Index) was used to evaluate the accuracy of the model. Higher C-Index values indicate more accurate prediction. The area under the receiver operating characteristic curve (AUC) was used to evaluate the discriminative ability of the nomogram. AUC values closer to 1 indicate better model discrimination (25, 26). The bootstrap method was used to re-sample the data 1,000 times and draw calibration curves to verify consistency between the predicted 1-year, 3-year, and 5-year CSS and actual survival. Better degrees of calibration reflect better coincidence between the survival probability predicted by the nomogram and the actual survival probability. Decision curve analysis (DCA) was used to assess the clinical practicability of the nomogram (27, 28). The clinical records of 53 patients with pathologically diagnosed EOC who underwent surgery in the Department of Gynecology at Shengjing Hospital of China Medical University from 2008 to 2012 were collected for external validation of the model. Moreover, all patients were regrouped into low- and high-risk groups based on the median risk score generated from the nomogram among the training cohort patients. Kaplan–Meier curves and the log-rank test were used to compare CSS between the two groups. The flow chart of study procedures is shown in **Figure 1**.

All statistical analyses were performed using SPSS Mac version 25.0 (Chicago, IL, USA) and R software version 3.6.0. Differences were considered significant if P < 0.05.

## Results

### Clinicopathological Characteristics of Patients

In this study, a total of 7129 EOC patients were included from the SEER database, including the training cohort (n=4992) and the validation cohort (n=2137). There was no difference in various indicators between the two groups (P < 0.05, **Table 1**). Most of the patients were white (83.25%), the majority AJCC stage of the patients were stage III (47.30%), the most histological grade was G3 and G4 (75.27%), the histological type was mainly serous carcinoma (67.05%), and 83.85% of the patients received postoperative chemotherapy. The mean survival time of all patients was 59.153 months (95%CI 58.381-59.926), and the 1-year, 3-year, and 5-year CSS rates were 91.9%, 72.1%, and 57.7%, respectively. Characteristics of patients are shown in **Table 1**.

**Table 1:** The clinicopathological characteristics and group comparison of 7129 patients with EOC.

Variables	n (%)	Mean survival time (95%CI) (months)	Training set [n (%) ]	Validation set [n (%)]	P^*^
Total	7129		4992	2137	
Year of diagnosis					0.439
2010-2011	2282 (32.0)	58.955 (57.772-60.139)	1620 (32.5)	662 (31.0)	
2012-2013	2339 (32.8)	46.301 (45.545-47.057)	1621 (32.5)	718 (33.6)	
2014-2015	2508 (35.2)	30.709 (30.334-31.084)	1751 (35.1)	757 (35.4)	
Age					0.655
19-53	2299 (32.2)	65.069 (63.815-66.323)	1596 (32.0)	703 (32.9)	
54-68	3228 (45.3)	59.182 (58.033-60.331)	2262 (45.3)	966 (45.2)	
69-	1602 (22.5)	50.624 (48.926-52.323)	1134 (22.7)	468 (21.9)	
Race					0.741
White	5935 (83.3)	59.067 (58.223-59.911)	4149 (83.1)	1786 (83.6)	
Black	395 (5.5)	52.028 (48.635-55.422)	278 (5.6)	117 (5.5)	
Asian	725 (10.2)	63.152 (60.771-65.534)	514 (10.3)	211 (9.9)	
American Indian	54 (0.8)	63.646 (54.989-72.303)	35 (0.7)	19 (0.9)	
Unknown	20 (0.3)	58.375 (53.562-63.188)	16 (0.3)	4 (0.2)	
Insure					0.399
Unknown	41 (0.6)	60.440 (51.196-69.683)	26 (0.5)	15 (0.7)	
Insured	6860 (96.2)	59.101 (58.313-59.890)	4813 (96.4)	2047 (95.8)	
Uninsured	228 (3.2)	59.940 (55.724-64.155)	153 (3.1)	75 (3.5)	
Marriage					0.423
Single	1409 (19.8)	61.666 (59.943-63.388)	1016 (20.4)	393 (18.4)	
Married	3987 (55.9)	60.399 (59.386-61.411)	2777 (55.6)	1210 (56.6)	
Widowed/Separated	773 (10.8)	51.456 (49.015-53.896)	535 (10.7)	238 (11.1)	
Divorced	730 (10.2)	55.656 (53.203-58.109)	507 (10.2)	223 (10.4)	
Unknown	230 (3.2)	60.114 (55.697-64.531)	157 (3.1)	73 (3.4)	
Laterality					0.875
Unilateral	3893 (54.6)	64.676 (63.678-65.674)	2736 (54.8)	1157 (54.1)	
Paired	66 (0.9)	48.422 (40.909-55.934)	46 (0.9)	20 (0.9)	
Bilateral	3170 (44.5)	52.893 (51.738-54.047)	2210 (44.3)	960 (44.9)	
Tumor size (mm)					0.354
No tumor	23 (0.3)	73.961 (64.510-83.411)	14 (0.3)	9 (0.4)	
<=62	2253 (31.6)	53.719 (52.313-55.124)	1559 (31.2)	694 (32.5)	
>=63	4853 (68.1)	61.623 (60.707-62.540)	3419 (68.5)	1434 (67.1)	
Preoperative serum CA125 level					0.812
Negative	802 (11.2)	73.282 (71.578-74.986)	565 (11.3)	237 (11.1)	
Borderline/positive	6327 (88.8)	57.318 (56.487-58.149)	4427 (88.7)	1900 (88.9)	
Surgery for primary lesions					0.809
No debulking	3004 (42.1)	68.482 (67.460-69.503)	2112 (42.3)	892 (41.7)	
Debulking	3967 (55.6)	51.902 (50.829-52.975)	2767 (55.4)	1200 (56.2)	
Pelvic exenteration	158 (2.2)	51.298 (46.187-56.409)	113 (2.3)	45 (2.1)	
Regional LN dissected					0.842
Undo	2588 (36.3)	50.023 (48.691-51.355)	1823 (36.5)	765 (35.8)	
1-3 Reg	914 (12.8)	55.243 (53.059-57.426)	639 (12.8)	275 (12.9)	
4 or more Reg	3627 (50.9)	66.448 (65.472-67.424)	2530 (50.7)	1097 (51.3)	
Grade					0.833
I	676 (9.5)	77.428 (75.942-78.913)	480 (9.6)	196 (9.2)	
II	1087 (15.2)	68.660 (66.972-70.348)	758 (15.2)	329 (15.4)	
III-IV	5366 (75.3)	54.831 (53.918-55.744)	3754 (75.2)	1612 (75.4)	
Histological types					0.825
Serous	4780 (67.1)	55.357 (54.410-56.304)	3331 (66.7)	1449 (67.8)	
Mucinous	442 (6.2)	69.537 (66.804-72.269)	319 (6.4)	123 (5.8)	
Endometrioid	1023 (14.3)	75.218 (73.841-76.594)	715 (14.3)	308 (14.4)	
Clear cell	562 (7.9)	64.183 (61.509-66.858)	403 (8.1)	159 (7.4)	
Transitional	36 (0.5)	65.188 (56.208-74.167)	26 (0.5)	10 (0.5)	
Epithelial-stromal	286 (4.0)	37.624 (33.581-41.667)	198 (4.0)	88 (4.1)	
AJCC Stage					1
I	1714 (24.0)	78.316 (77.473-79.159)	1200 (24.0)	514 (24.1)	
II	753 (10.6)	69.403 (67.339-71.468)	528 (10.6)	225 (10.5)	
III	3372 (47.3)	53.253 (52.118-54.388)	2361 (47.3)	1011 (47.3)	
IV	1290 (18.1)	42.603 (40.786-44.421)	903 (18.1)	387 (18.1)	
Residual lesion size					0.215
No Residual lesion	5014 (70.3)	65.611 (64.755-66.468)	3507 (70.3)	1507 (70.5)	
<=1cm	1035 (14.5)	46.838 (44.861-48.814)	720 (14.4)	315 (14.7)	
>1cm	542 (7.6)	42.620 (39.994-45.247)	399 (8.0)	143 (6.7)	
Residual unknown	538 (7.5)	42.329 (39.579-45.080)	366 (7.3)	172 (8.0)	
LN Positive					0.795
Neg/Unknown	5493 (77.1)	61.101 (60.236-61.965)	3854 (77.2)	1639 (76.7)	
<=3	987 (13.8)	54.427 (52.286-56.567)	691 (13.8)	296 (13.9)	
>=4	649 (9.1)	49.781 (47.199-52.362)	447 (9.0)	202 (9.5)	
Radiotherapy					0.517
None/Unknown	7062 (99.1)	59.272 (58.497-60.048)	4948 (99.1)	2114 (98.9)	
Yes	67 (0.9)	46.319 (38.513-54.125)	44 (0.9)	23 (1.1)	
Neoadjuvant chemotherapy					0.224
None/Unknown	6468 (90.7)	60.936 (60.137-61.735)	4515 (90.4)	1953 (91.4)	
Yes	661 (9.3)	41.522 (39.136-43.908)	477 (9.6)	184 (8.6)	
Chemotherapy					0.42
None/Unknown	1151 (16.1)	65.466 (63.618-67.314)	794 (15.9)	357 (16.7)	
Yes	5978 (83.9)	57.878 (57.035-58.722)	4198 (84.1)	1780 (83.3)	
Organ metastasis					
bone					1
None/Unknown	7110 (99.7)	59.262 (58.490-60.035)	4979 (99.7)	2131 (99.7)	
Yes	19 (0.3)	18.033 (10.963-25.103)	13 (0.3)	6 (0.3)	
brain					0.329
None/Unknown	7124 (99.9)	59.172 (58.399-59.945)	4987 (99.9)	2137 (100)	
Yes	5 (0.1)	30.200 (15.732-61.034)	5 (0.1)	0 (0.0)	
liver					0.824
None/Unknown	6813 (95.6)	59.888 (59.105-60.672)	4773 (95.6)	2040 (95.5)	
Yes	316 (4.4)	43.198 (39.375-47.021)	219 (4.4)	97 (4.5)	
lung					0.635
None/Unknown	6898 (96.8)	59.841 (59.060-60.621)	4834 (96.8)	2064 (96.6)	
Yes	231 (3.2)	38.185 (34.157-42.214)	158 (3.2)	73 (3.4)	

^*^Comparing the distribution of the training and validation group.

### Nomogram Construction

#### Univariate Analysis and LASSO Analysis

**Figure 2:**
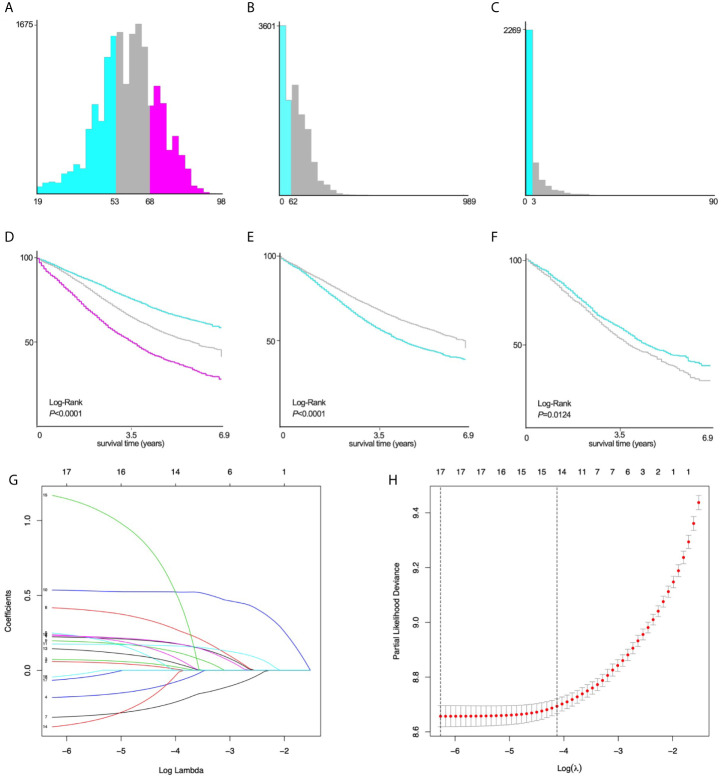
X-tile stratification and LASSO analysis. Histograms based on the appropriate cut-off points of **(A)** age (≤53 years vs. 54~68 years vs. ≥69 years), **(B)** tumor size (≤62mm vs. ≥63mm), and **(C)** the number of positive lymph nodes (≤3 vs. ≥4). The Kaplan–Meier curves for CSS in patients with EOC stratified according to **(D)** age (P<0.0001), **(E)** tumor size (P<0.0001), **(F)** positive lymph nodes (P=0.0124). **(G)** LASSO coefficient profiles of 17 variables for CSS; **(H)** LASSO analysis identified 17 variables for CSS. LASSO, least absolute shrinkage and selection operator; CSS, cancer-specific survival; EOC, epithelial ovarian cancer.

The variables are stratified according to the cut-off points which were ascertained by X-tile software v3.6.1: age: ≤53 years, 54~68 years and ≥69 years; tumor size: ≤62mm and ≥63mm; number of positive lymph nodes: ≤3 and ≥4 (**Figures 2A–F**).

Univariate Log-rank χ2 test was performed and the factors with *P* < 0.01 were reserved. Among the 22 variables, year, race, insurance, radiation, and brain metastasis with *P* > 0.01 are excluded (**Table 2**). Lasso Cox regression analysis was performed on the remaining 17 variables, and the results showed that there were no further excluded factors (**Figures 2G, H**).

**Table 2:** Univariate and multivariate Cox regression analysis.

Variables	Univariate analysis		Multivariate analysis	
	HR (95% CI)	*P*	HR (95% CI)	*P*
Year of diagnosis	0.989 (0.921-1.062)	0.76	–	
2010-2011				
2012-2013				
2014-2015				
Age	1.487 (1.391-1.591)	<0.001^**^		
19-53			Reference	
54-68			1.190 (1.048-1.352)	0.007^*^
69-			1.527 (1.320-1.766)	1.15e-08^***^
Race	0.951 (0.883-1.024)	0.182	–	
White				
Black				
Asian				
American Indian				
Unknown				
Insure	0.938 (0.723-1.217)	0.629	–	
Unknown				
Insured				
Uninsured				
Marriage	1.120 (1.068-1.176)	<0.001^**^		
Single			Reference	
Married			0.922 (0.802-1.060)	0.252
Widowed/Separated			1.215 (1.010-1.461)	0.039^*^
Divorced			1.172 (0.972-1.413)	0.096
Unknown			1.048 (0.758-1.449)	0.778
Laterality	1.397 (1.329-1.469)	<0.001^**^		
Unilateral			Reference	
Paired			1.058 (0.689-1.624)	0.798
Bilateral			1.159 (1.037-1.295)	0.009^*^
Tumor size (mm)	0.697 (0.631-0.768)	<0.001^**^		
No tumor			Reference	
<=62			0.989 (0.244-4.001)	0.987
>=63			0.805 (0.199-3.252)	0.76
Preoperative serum CA125 level	2.769 (2.214-3.464)	<0.001^**^		
Negative			Reference	
Borderline/positive			1.303 (1.030-1.647)	0.027^*^
Surgery for primary lesions	2.218 (2.024-2.431)	<0.001^**^		
No debulking			Reference	
Debulking			1.286 (1.138-1.453)	5.49e-05^**^
Pelvic exenteration			1.222 (0.886-1.687)	0.222
Regional LN dissected	0.634 (0.601-0.669)	<0.001^**^		
Undo			Reference	
1-3 Reg			0.881 (0.747-1.038)	0.13
4 or more Reg			0.562 (0.483-0.655)	1.42e-13^***^
Grade	2.507 (2.218-2.833)	<0.001^**^		
I			Reference	
II			2.422 (1.641-3.574)	8.48e-06^***^
III-IV			3.194 (2.187-4.662)	1.81e-09^***^
Histological types	0.936 (0.898-0.976)	0.002^*^		
Serous			Reference	
Mucinous			3.145 (2.282-4.335)	2.63e-12^***^
Endometrioid			1.054 (0.823-1.350)	0.678
Clear cell			2.049 (1.645-2.551)	1.46e-10^***^
Transitional			0.882 (0.394-1.975)	0.761
Epithelial-stromal			3.363 (2.762-4.094)	<2e-16^***^
AJCC Stage	2.214 (2.084-2.351)	<0.001^**^		
I			Reference	
II			2.623 (1.893-3.634)	6.75e-09^***^
III			5.093 (3.831-6.770)	<2e-16^***^
IV			6.787 (5.005-9.204)	<2e-16^***^
Residual lesion size	1.552 (1.487-1.619)	<0.001^**^		
No Residual lesion			Reference	
<=1cm			1.331 (1.162-1.525)	3.66e-05^***^
>1cm			1.599 (1.364-1.874)	7.32e-09^***^
Residual unknown			1.562 (1.327-1.839)	8.25e-08^***^
LN Positive	1.292 (1.205-1.385)	<0.001^**^		
Neg/Unknown			Reference	
<=3			1.155 (0.975-1.369)	0.096
>=4			1.617 (1.330-1.967)	1.43e-06^***^
Radiotherapy	1.738 (1.130-2.673)	0.012^*^		
None/Unknown				
Yes				
Neoadjuvant chemotherapy	2.394 (2.092-2.740)	<0.001^**^		
None/Unknown			Reference	
Yes			1.145 (0.988-1.328)	0.073
Chemotherapy	1.578 (1.350-1.844)	<0.001^**^		
None/Unknown			Reference	
Yes			0.614 (0.521-0.724)	6.93e-09^***^
Organ metastasis				
bone	5.978 (3.302-10.823)	<0.001^**^		
None/Unknown			Reference	
Yes			3.617 (1.927-6.791)	6.33e-05^**^
brain	3.489 (1.124-10.831)	0.031^*^		
None/Unknown				
Yes				
liver	2.273 (1.876-2.756)	<0.001^**^		
None/Unknown			Reference	
Yes			1.075 (0.867-1.334)	0.51
lung	2.601 (2.108-3.208)	<0.001^**^		
None/Unknown			Reference	
Yes			1.073 (0.852-1.352)	0.55

*P<0.05, **P<0.001, ***P<5.00e–05.

#### Multivariate Analysis

All the 17 variables conforming to the analysis were included in the multivariate Cox analysis (**Table 2**). The variables with *P*<5.00e-05 were identified as independent prognostic factors, including age, regional lymph nodes dissected, lymph nodes positive, residual lesions size, histological grade, histologic types, AJCC stage and chemotherapy.

#### Nomogram Construction

**Figure 3:**
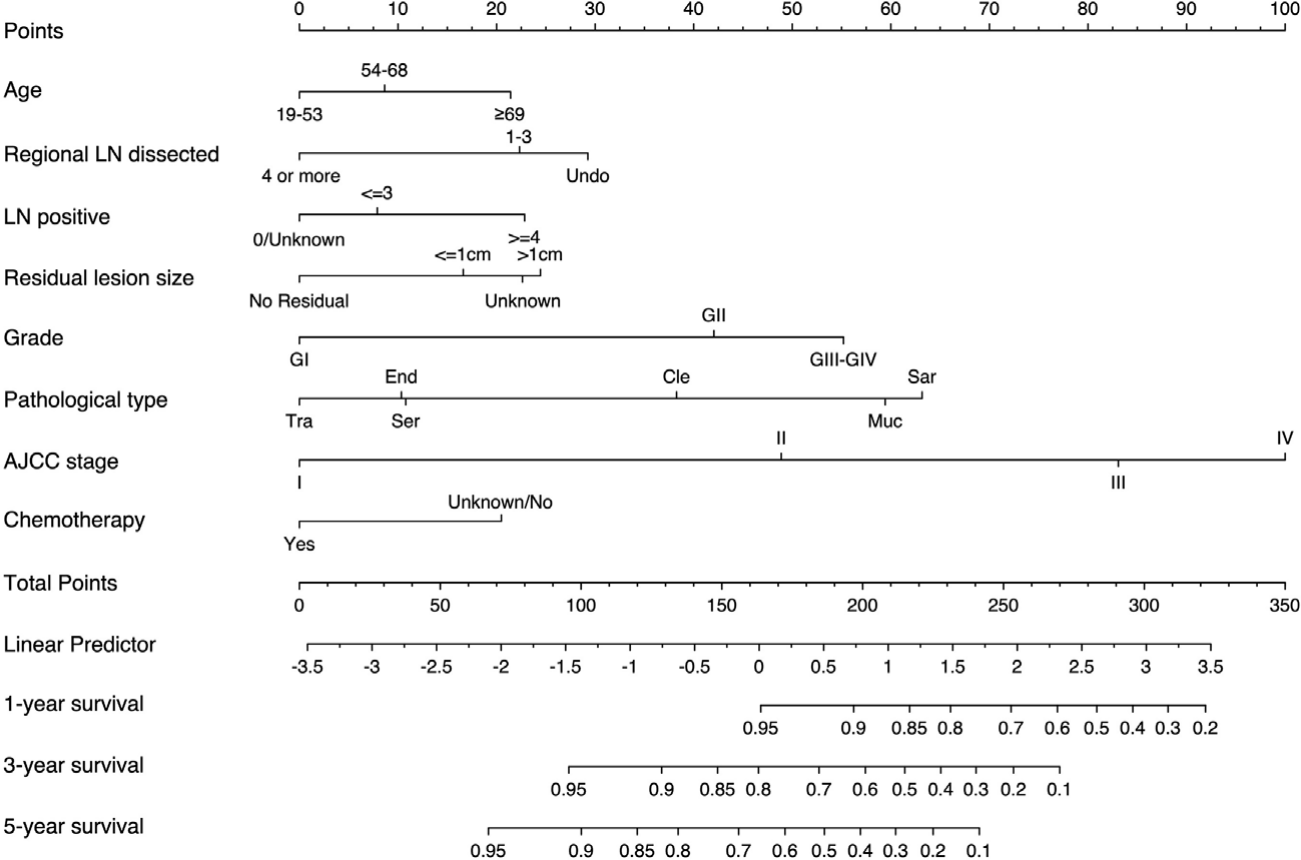
Nomogram for predicting 1-, 3-, and 5-year CSS in adult patients who underwent primary surgery for EOC. LN, lymph node; AJCC, American Joint Commission on Cancer; CSS, cancer-specific survival; EOC, epithelial ovarian cancer; Ser, serous; Muc, mucinous; End,endometrioid; Cle, clear cell adenocarcinoma; Tra, transitional cell tumor; Sar, epithelial-stromal.

We established the nomogram based on the above independent prognostic factors for CSS. The nomogram was displayed for predicting the 1-, 3-, 5-year CSS (**Figure 3**). The different subtypes of each independent prognostic factor were projected onto the score scale to obtain the score for each item. The scores corresponding to independent prognostic factors were added to obtain the total score. A vertical line was drawn down on the total score scale to obtain the 1-, 3-, 5-year CSS. The higher the total score, the worse the prognosis. According to the patient information, this nomogram can obtain the individualized prediction of CSS, which improves the accuracy and efficiency of the prediction.

### Nomogram Validation

#### Internal Validation

We validated the model internally in the training cohort and the validation cohort.

In the training cohort, the C-index of the nomogram (0.763 [95%CI 0.751-0.775]) was higher than the AJCC stage (0.687 [95%CI 0.675-0.699]) and histological grade (0.590 [95%CI 0.581-0.599]). In the validation cohort, the C-index of the nomogram (0.750 [95%CI 0.731-0.769]) was also higher than the AJCC stage (0.672 [95%CI 0.653-0.691]) and histological grade (0.581 [95%CI 0.567-0.595]). In addition, we found that the C-index values of our nomogram in the training cohort and the validation cohort were both higher than that of J.N. Barlin et al.’s study (0.714) and M.J. Rutten et al.’s study (0.710 [95% CI 0.690-0.740]) (**Table 3**).

**Table 3:** Comparison of C-indexes in EOC patients.

		C index (95%CI)
Training cohort	Nomogram	0.763 (0.751-0.775)
	AJCC stage	0.687 (0.675-0.699)
	Histological grade	0.590 (0.581-0.599)
Validation cohort	Nomogram	0.750 (0.731-0.769)
	AJCC stage	0.672 (0.653-0.691)
	Histological grade	0.581 (0.567-0.595)
J.N. Barlin et al. (17)	Nomogram	0.714
	stage	0.620
M.J. Rutten et al. (18)	Nomogram	0.71 (0.69-0.74)

**Figure 4:**
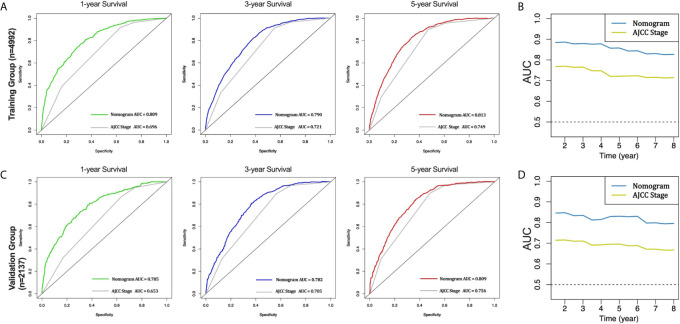
AUC curves of the nomogram and AJCC stage in prediction of prognosis in the training and validation cohorts. AUC curves of the nomogram and AJCC stage in prediction of prognosis at 1-, 3- and 5-year point in the training cohort **(A)**. Time dependent AUC curves of the nomogram and AJCC stage from 0.5 year to 8 year in the training cohort **(B)**. AUC curves of the nomogram and AJCC stage in prediction of prognosis at 1-, 3- and 5-year point in the validation cohort **(C)**. Time dependent AUC curves of the nomogram and AJCC stage from 0.5 year to 8 year in the validation cohort **(D)**.

Furthermore, the AUCs of the nomogram were higher than AJCC stage in both training (1-year AUC: 0.809 vs. 0.696, 3-year AUC: 0.790 vs. 0.721, 5-year AUC: 0.813 vs. 0.749, **Figure 4A**) and validation (1-year AUC: 0.785 vs. 0.653, 3-year AUC: 0.782 vs. 0.705, 5-year AUC: 0.809 vs. 0.756, **Figure 4C**) cohorts for 1-, 3- and 5-year CSS, respectively. In addition, we further compared the nomogram with AJCC stage based on the time-dependent AUCs from the half a year to the eighth year, and found that the nomogram performs obviously better in both the training and the validation cohorts, respectively (**Figures 4B, D**). These results indicate that the nomogram has better degree of discrimination than traditional AJCC stage in both the training and validation cohorts.

**Figure 5:**
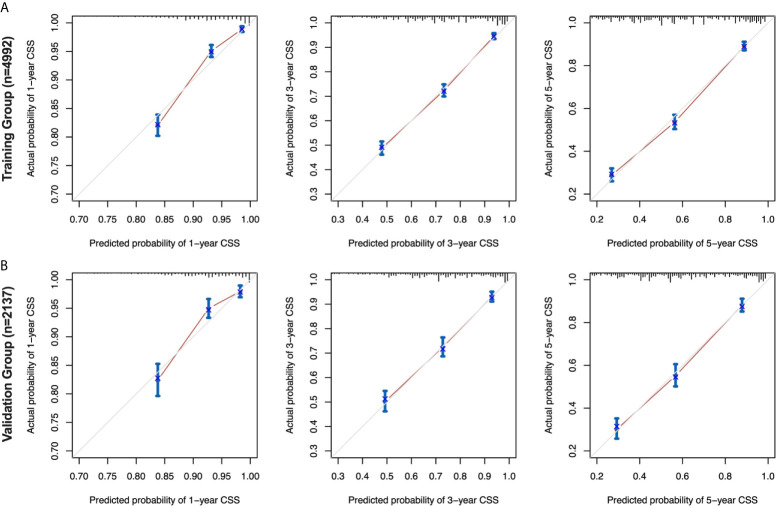
Calibration curves for the nomogram in the training and validation cohorts. 1-, 3-, and 5-year calibration curves **(A)** for the CSS nomogram in the training cohort of patients with EOC (bootstrap = 1,000 repetitions). 1-, 3-, and 5-year calibration curves **(B)** for the CSS nomogram in the validation cohort of patients with EOC (bootstrap = 1,000 repetitions). EOC, epithelial ovarian cancer; CSS, cancer-specific survival.

The calibration curves for the 1-, 3-and 5-year CSS were all close to the gray line of the ideal case, which indicated that there was high degree of consistency between the actual survival probability and the prediction in both the training cohort (**Figure 5A**) and the validation cohort (**Figure 5B**).

**Figure 6:**
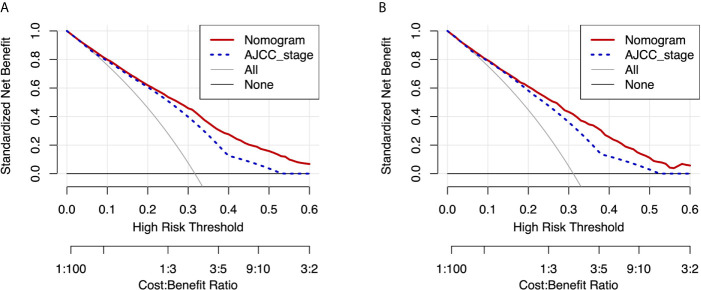
DCA curves of the nomogram and AJCC stage for CSS in the training and validation cohorts. DCA curves of the nomogram and AJCC stage for CSS in both the training cohort **(A)** and validation cohort **(B)**. DCA, decision curve analysis; AJCC, American Joint Commission on Cancer; CSS, cancer-specific survival.

Moreover, DCA curves in the training and validation cohorts also showed favorable prediction effects and had better clinical application value than the AJCC stage (**Figure 6**).

#### External Validation

External data verification of our nomogram model was performed. A total of 53 patients with primary EOC who underwent surgery in the Department of Gynecology, Shengjing Hospital of China Medical University from 2008 to 2012 were collected, and all of them qualified for inclusion.

The mean age of all patients was 58.2 ± 1.24 years old (42-78 years old, median age was 58 years old). In the stages, the proportion of stage I-II was 43.3%. In the differentiation, the proportion of high, middle and low differentiation was 11.3%, 45.3%, and 43.4%, respectively. Among the histological types, the proportions of serous, mucinous, endometrioid, clear cell adenocarcinoma, transitional and epithelial-stromal were 45.3%, 17.0%, 9.4%, 13.2%, 3.8%, and 11.3%, respectively. The percentage of lymph node resection with 1-3 or more than 4 regions was 79.2% and 15.1%. Among the positive lymph nodes, the number of lymph nodes ≤ or ≥4 were 18.9% and 11.3%, respectively. Among the residual lesions, no residual lesions, residual lesions ≤ 1cm and residual lesions ≥ 1cm were 71.7%, 11.3%, and 11.3%, respectively. 81.1% of the patients received postoperative chemotherapy (**Supplementary Table 1**).

**Figure 7:**
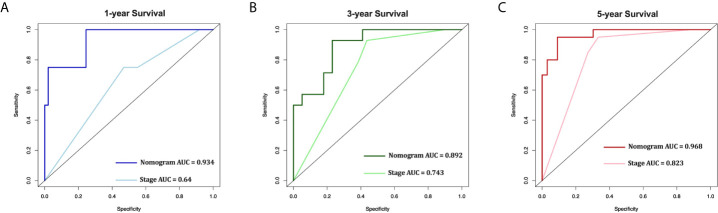
External validation of the nomogram compared with AJCC stage in 53 cases of EOC from Shengjing Hospital. AUC curves of the nomogram and AJCC stage in the prediction of prognosis at the 1- **(A)**, 3- **(B)**, and 5-year **(C)** points.

In the 53 patients, the C-index of the nomogram (0.920 [95%CI 0.875-0.965]) was higher than the AJCC stage (0.758 [95%CI 0.672-0.844]). Furthermore, the AUCs of the nomogram were significantly higher than AJCC stage (1-year AUC: 0.934 vs. 0.640, 3-year AUC: 0.892 vs. 0.743, 5-year AUC: 0.968 vs. 0.823, **Figure 7**) for 1-, 3- and 5-year CSS.

The above internal and external verification results indicate that our nomogram has better performance.

### Patient Risk Stratification

**Figure 8:**
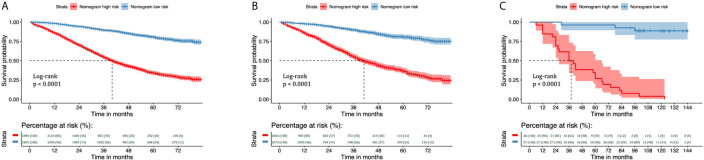
Kaplan–Meier curves of CSS for risk classification based on the nomogram scores in the training and validation cohorts. Kaplan–Meier curves of CSS for risk classification based on the nomogram scores **(A)** in the training cohort, **(B)** in the validation cohort, and **(C)** in the 53 patients with EOC treated at Shengjing Hospital. CSS, cancer-specific survival; EOC, epithelial ovarian cancer.

We divided the training and validation cohorts and the 53 patients into high-and low-risk groups based on the cutoff values separately. Kaplan-Meier survival analysis showed favorable CSS in the low-risk group compared with the high-risk group. Low-risk patients’ CSS rates were all higher than those of high-risk patients. (**Figure 8**, all P < 0.001).

## Discussion

Ninety percent of OC cases are epithelial, the vast majority of which occur in adults and are associated with poor prognosis. Reliable determinations of prognosis for adult EOC remain a difficult problem for clinicians. However, nomograms can be used to evaluate individual survival prognosis according to disease characteristics with high accuracy, which can aid in clinical decision-making for patients with various types of tumors (29–31). Moreover, nomograms have been significantly better at judging prognosis than traditional AJCC stage and clinician experience. At present, there is no reliable, large sample-based, real world tool for evaluating postoperative prognosis among adult patients with EOC. Therefore, using data from the SEER database, the present study aimed to evaluate prognostic factors for 1-, 3-, and 5-year CSS and establish an appropriate individualized nomogram for predicting prognosis in adult patients with EOC following primary surgery. Our internal and external validation results revealed a perfect prediction effect, suggesting that the nomogram can be highly useful in clinical situations.

Our study identified eight independent prognostic factors for CSS, including age, number of regional lymph nodes dissected, number of positive lymph nodes, residual lesion size, histological grade, histological type, AJCC stage, and chemotherapy. In general, older patients are more likely to have poorer survival outcomes. Kim et al. (32) conducted a retrospective analysis of 1,236 patients with EOC, reporting that an age of 66 years was the most significant cut-off for defining the effect of old age with independent prognostic power (HR=1.45; 95% confidence interval=1.04–2.03; p=0.027). In their survival analysis, patients aged ≥66 years had significantly worse overall survival than younger individuals (56 months vs. 87 months; p=0.006). In the present study, age ≥69 years was an independent risk factor for CSS in patients with EOC after primary surgery, and the results were basically consistent.

The degree of cell differentiation or tumor histological grade has been considered to influence the biological behavior of the tumor and patient survival. Grade 1 tumors are associated with higher 3-year disease-specific survival rates (96.4%) than grade 2 (92.4%) or 3 (82.0%) tumors (P<0.001) in patients with early-stage EOC (33). In the present study, tumor histological grade was classified into well-differentiated (G1), moderately differentiated (G2), poorly differentiated (G3) and undifferentiated (G4). Mean CSS for G1, G2, and G3–G4 tumors was 77.428 months (95% CI: 75.942–78.913), 68.660 months (95%CI: 75.942–78.913), and 54.831 months (95% CI: 75.942–78.913), respectively. Multivariate analysis suggested that histological grade was an independent risk factor for patients with EOS, which further indicated that histological tumor grades are associated with worse prognosis among patients with EOC.

The effect of histological types of EOC on prognosis remains controversial. In one study involving 9,491 patients with EOC, 10-year survival rates were better among patients with mucinous, endometrioid, or clear-cell carcinoma than among those with serous carcinoma, although 10-year survival was worse for carcinosarcoma than for serous carcinoma (34). However, some scholars have proposed that long-term survival is worse among patients with clear-cell carcinoma than among those with serous carcinoma, or that there is no significant difference in survival between the two (35, 36). Among all six histological types observed in this study, the epithelial-stromal type (including adenosarcoma and carcinosarcoma) was associated with the shortest mean survival time (37.624 months [95% CI: 33.581–41.667]) and had the worst prognosis.

Previous research has indicated that tumor stage is the most prominent independent factor for progression-free survival (PFS) and OS (37). In the present study, mean survival times (months) for AJCC stages I, II, III, and IV were 78.316 (77.473–79.159), 69.403 (67.339–71.468), 53.253 (52.118–54.388), and 42.603 (40.786–44.421), respectively, and the differences were statistically significant (P0.002). Multivariate analysis revealed that AJCC stage was an independent prognostic factor for EOC, indicating that clinical stage was an important factor affecting the prognosis of EOC. This result suggests that, while focusing on improving clinical efficacy, early detection, early diagnosis, and early treatment are necessary to improve long-term outcomes among patients with EOC.

The most effective tumor cytoreductive surgeries are those in which there are no visible lesions remaining after the initial surgery. Residual tumor volume has been identified as an independent predictor of prognosis in patients with EOC (38). Data from three European prospective randomized trials (AGO-OVAR 3, AGO-OVAR 5, and AGO-OVAR 7) demonstrated that R0 resection was associated with significantly longer median overall survival (R0 resection, 99.1 months vs. <1 cm residual disease, 36.2 months vs. >1 cm residual disease, 29.6 months; P<0.0001) (39, 40). In our study, the mean survival time (months) of patients without residual lesions was 65.611, which was significantly higher than that of patients with residual lesions (46.838 for residual lesions ≤1 cm vs. 42.62 for residual lesions with >1 cm vs. 42.329 for residual lesions of unknown dimensions). Our multivariate analysis revealed that postoperative residual lesion size was an independent risk factor affecting the prognosis of patients with EOC: Satisfactory tumor cell reduction was associated with better prognosis than unsatisfactory tumor cell reduction, highlighting the importance of R0 resection.

We also analyzed the influence of the number of regional lymph nodes dissected and the number of positive lymph nodes on prognosis. The issue of lymph node dissection for OC remains controversial. A multi-center prospective randomized controlled trial (41) reported that systematic pelvic and paraaortic lymphadenectomy in patients with advanced OC who had undergone intraabdominal macroscopically complete resection and had normal lymph nodes both before and during surgery was not associated with longer OS or PFS than no lymphadenectomy. The authors also reported that systematic pelvic and paraaortic lymphadenectomy was associated with a higher incidence of postoperative complications. As a result, the researchers suggested that systemic lymphadenectomy should not be performed in patients with advanced OC who are clinically assessed with negative lymph nodes and have no residual lesions visible to the naked eye. This recommendation was adopted by the National Cancer Care Alliance (NCCA) guidelines in 2019. However, our analysis indicated that the number of regional lymph nodes resected was an independent protective factor for the prognosis of patients with EOC, and that patients with four or more regional lymph nodes resected had the best prognosis. Moreover, lymph node positivity was an independent risk factor for EOC prognosis, and patients with ≥4 positive lymph nodes had the worst prognosis. Thus, our findings highlight the importance of systemic lymphadenectomy for prognosis in patients with EOC. Further studies are required to compare the influence of the number of regional lymph nodes resected and the number of positive lymph nodes on prognosis in patients with early-stage and advanced EOC.

In 2018, the National Comprehensive Cancer Network (NCCN) still proposed that, among patients with EOC who can endure surgery, most will require postoperative chemotherapy after standardized transabdominal comprehensive staging surgery and tumor reduction surgery, with the aim of reducing the recurrence of EOC or treating residual lesions. Platinum combined with paclitaxel is the “gold standard” first-line chemotherapy regimen. In our study, we noted that chemotherapy was significantly associated with CSS, indicating its value in improving survival outcomes.

Based on the eight independent prognostic factors identified above, we constructed a nomogram to evaluate CSS of EOC in adults. It is well known that when the C-index and AUC exceed 0.7, the model has good predictive ability. In our study, the C-Index and AUC of the nomogram were both higher than 0.7, and both were higher than those for the AJCC staging system, indicating a better prediction effect. The calibration curve also revealed good agreement between the nomogram prediction and actual survival. Analysis of the DCA curve further confirmed that the nomogram exhibited better performance than the AJCC staging system. In addition, based on the nomogram, we developed a risk stratification system that allowed for clear division of all patients into two risk groups. The differences in the survival curves of the different risk subgroups were statistically significant.

Previous researchers have also established nomograms for predicting CSS in patients with EOC following surgery [17,18]. However, these studies were conducted with few patients at single centers, and the variables in their nomograms and the SEER database are not completely consistent, making it difficult to directly compare the nomograms. However, our C-index was higher (training cohort: 0.763 [95% CI: 0.751–0.775] vs. internal validation cohort: 0.750 [95% CI: 0.731–0.769] vs. 53 external validation patients: 0.920 [95% CI: 0.875–0.965]) than those reported by Barlin et al. (0.714) and Rutten et al. (0.710) [95% CI: 0.690–0.740]). Importantly, our model is based on a large-sample database in the real world, making our findings more reliable.

More importantly, the previous nomograms have rarely been externally validated. In the present study, external validation was performed using data from 53 eligible patients with EOC. The C-index and AUC results also indicated that the nomogram exhibited excellent performance, which was better than that for the AJCC staging system. Therefore, to our knowledge, our nomogram is currently the most optimal and directly applicable model for predicting CSS in adults who have undergone primary surgery for EOC.

Our study also has some limitations. First, selection bias is inevitable due to the retrospective nature of the study. Second, there is a lack of some important information in the SEER database. For example, the specific pathological types of epithelial carcinoma are not always mentioned, which leads to a certain extent of bias in the data analysis. Third, there are many potentially important factors affecting postoperative outcomes, such as preoperative examinations (positron emission tomography [PET]/computed tomography [CT], serum human epididymis protein 4 (HE4), Risk of Ovarian Malignancy Algorithm [ROMA] score, etc.), ECOG performance status, specific preoperative comorbidities (ascites, intestinal obstruction, etc.), operative time, and occurrence of serious postoperative complications (pulmonary infarction, infection of lymphatic cyst, etc.), none of which could be included in this study.

## Conclusion

The large-scale SEER database was used to construct a nomogram that could accurately evaluate 1-, 3-, and 5- year CSS for adult patients with EOC following surgery. The nomogram was internally validated using the SEER database and externally validated in patients with EOC treated at Shengjing Hospital of China Medical University. A risk stratification system was established based on the risk score generated by the nomogram. To our knowledge, our nomogram is currently the most optimal and directly applicable model for predicting CSS in adult patients with EOC following surgery in clinical practice.

## Data Availability Statement

The datasets presented in this article are not readily available because No. Requests to access the datasets should be directed to LZ, medecin@126.com.

## Ethics Statement

The studies involving human participants were reviewed and approved by Shengjing Hospital of China Medical University. The patients/participants provided their written informed consent to participate in this study.

## Author Contributions

XL and LZ designed research and wrote manuscript. JG and LY involved in data collection and data statistical analysis. LZ provided fund support and critically reviewed the manuscript. All authors contributed to the article and approved the submitted version.

## Funding

The present study was supported by 345 Talent Project of Shengjing Hospital.

## Conflict of Interest

The authors declare that the research was conducted in the absence of any commercial or financial relationships that could be construed as a potential conflict of interest.
