# Supplementary material for: A Novel Clinical Nomogram for Predicting Cancer-Specific Survival in Adult Patients After Primary Surgery for Epithelial Ovarian Cancer: A Real-World Analysis Based on the Surveillance, Epidemiology, and End Results Database and External Validation in a Tertiary Center
Source: Front Oncol. 2021 Apr 20;11:670644. doi: 10.3389/fonc.2021.670644 (PMC8093627; doi:10.3389/fonc.2021.670644)
Supplement: Supplementary file 1 [file Table_1.docx]

**Supplementary table 1. The clinicopathological characteristics and mean survival time of 53 patients with EOC in our hospital**

| Variables | All patients n (%) | | Mean survival time 95%CI (month) |
| --- | --- | --- | --- |
| Total | 53 |  |  |
| Age |  |  |  |
| 19-53 | 17 (32.1) |  | 87.353 (65.501-109.205) |
| 54-68 | 27 (50.9) |  | 92.726 (72.790-112.663) |
| 69- | 9 (17.0) |  | 80.792 (76.649-104.805) |
| Regional LN dissected |  |  |  |
| Undo | 3 (5.7) |  | 25.000 (4.537-45.463) |
| 1-3 Reg | 42(79.2) |  | 89.950 (74.562-105.337) |
| 4 or more Reg | 8 (15.1) |  | 109.500 (81.784-137.216) |
| LN positive |  |  |  |
| Neg/Unknown | 37 (69.8) |  | 104.489 (87.319-121.660) |
| <=3 | 10 (18.9) |  | 67.600 (48.244-86.956) |
| >=4 | 6 (11.3) |  | 41.667 (26.170-57.163) |
| Grade |  |  |  |
| I | 6 (11.2) |  | 87.833 (60.697-114.970) |
| II | 24 (44.4) |  | 85.708 (64.681-106.736) |
| III | 23 (43.4) |  | 87.766 (67.562-107.970) |
| Pathologic types |  |  |  |
| Ser/Muc/End | 38 (71.7) |  | 92.773 (76.485-109.062) |
| Cel/Tra/Sar | 15 (28.3) |  | 84.867 (57.872-111.862) |
| Stage |  |  |  |
| I-II | 23 (43.4) |  | 135.814 (123.754-147.873) |
| III-IV | 30 (56.6) |  | 55.967 (43.077-68.857) |
| Residual lesion size |  |  |  |
| No Res | 38 (71.8) |  | 105.598 (90.155-121.041) |
| <=1cm | 6 (11.3) |  | 62.000 (36.968-87.032) |
| >1cm | 6 (11.3) |  | 50.833 (28.290-73.377) |
| Res Unknown | 3 (5.6) |  | 15.000 (9.120-20.880) |
| Chemotherapy |  |  |  |
| No | 10 (18.9) |  | 61.150 (38.989-83.311) |
| Yes | 43 (81.1) |  | 95.803 (80.216-111.391) |
